# Delayed recovery following myasthenic exacerbation and subsequent endplate-protecting treatment

**DOI:** 10.1007/s10072-025-08617-6

**Published:** 2026-02-04

**Authors:** Anna Mück, Sonja Genau, Julia Emde, Christoph Best, Steffen Pfeuffer, Heidrun H. Krämer

**Affiliations:** 1 Department of Neurology, Justus Liebig University, Klinikstrasse 33, D-35392 Giessen, Germany; 2 Department of Neurology, Philipps-University, Marburg, Germany

**Keywords:** Myasthenia gravis, Myasthenic exacerbation, Endplate-protecting treatment, Real-world evidence

## Abstract

Endplate-protecting therapies have improved treatment of myasthenia gravis and indicate a rapid onset of their treatment effect. Currently, these substances can be used following myasthenic exacerbation (ME). Clinical worsening due to ME however often results in prolonged recovery. We here evaluated the treatment courses of patients having received endplate-protecting treatment following ME. Among 12 patients with ME described here, seven received endplate-protecting therapies. Our findings indicate that acceptable recovery usually takes nine months. In the lights of recent trials for treatment of myasthenia, our findings implicate that the effectiveness of novel substances should be judged only after sufficient observation periods.

## Introduction

In myasthenia gravis (MG), neuromuscular transmission is disturbed by antibody-mediated complement-dependent endplate-degradation. Treatment involves cholinesterase inhibitors, corticosteroids and immunosuppressants.

Novel “endplate-protecting treatments” (EPT), including complement inhibitors (C5I) and neonatal Fc receptor inhibitors (FcRnI) have shown rapid alleviation of symptoms by directly inhibiting endplate-degradation to improve symptoms [1].

However, ME recovery can take up to 12 months following standard-of-care (SOC) treatment [2]. We here describe the recovery in a subgroup of MG patients from our prospective cohort receiving EPT or SOC.

## Methods

### Patients and outcome measures

MG Patients were enrolled in our prospective cohort since 2022 following diagnosis according to international guidelines. Here, we included patients with: (I) ME with increased quantitative myasthenia gravis (QMG) score of ≥ 5 points requiring rescue therapy; (II) acetylcholine receptor antibodies; (III) thymectomy within five years of initial manifestation; (IV) at least 12 months of follow-up. All data were collected prospectively. We excluded patients with antibodies to muscle-specific kinase or lipoprotein receptor-related protein 4. QMG and MG-ADL scores were assessed upon regular visits as well as the patient-acceptable symptom state (PASS) [3]. Patients with SOC versus EPT were described separately.

## Results

Of 118 prospective MG patients, 93 had seropositive MG, and 20 experienced ME from 01/2022 to 12/2023. Of those, 12 were seropositive for acetylcholine receptor autoantibodies and underwent early thymectomy. In all patients, this was the very first ME. Due to ME, three of the patients required admission to intensive care unit. Two of them required intubation and ventilation due to respiratory insufficiency.

Triggers for ME were: reduction or interruption of DMT (SOC: 4/5, EPT: 4/7), infections (SOC: 1/5, EPT 2/5) or unknown (EPT: 1).

Table 1 depicts their baseline demographics.

**Table 1:** Baseline parameters of the included patients antibody testing includes the following autoantibodies: AChR, MuSK, LRP4, Titin and SOX1. Among infection resulting in ME, we observed one case of COVID-19, one case of influenza and one case of bacterial pneumonia. Abbreviations: MGFA: myasthenia Gravis foundation association; DMT: disease-modifying treatment; achr: acetylcholine receptor; ME: myasthenic exacerbation

	Standard-of-care (*n* = 5)	C5I/FcRnI therapy (*n* = 7)
Age, yrs, median (range)	*70 (37–84)*	*56 (35–72)*
Female patients (%)	*3 (60)*	*4 (57)*
Disease duration, months, median (range)	*84 (6–92)*	*68 (5–107)*
Baseline MGFA class:		
- II	*1 (20)*	*1 (14)*
- III	*3 (60)*	*4 (57)*
- IV	*1 (20)*	*2 (29)*
Number of previous DMT, median (range)	*1 (0–4)*	*1 (1–5)*
Body weight, kg, median (range)	*65 (58–82)*	*75 (57–170)*
Patients with oral corticosteroids at baseline (%)	*1 (20)*	*4 (57)*
Antibody status (%):		
- AChR^+^	*2 (40)*	*5 (71)*
- AChR^+^Titin^+^	*3 (60)*	*2 (29)*
Presence of thymoma upon thymectomy (%)	*0 (0)*	*1 (14)*
Reason for ME		
- reduction or interruption of DMT	*4 (80)*	*4 (57)*
- infection	*1 (20)*	*2 (29)*
- no obvious reason	*0 (0)*	*1 (14)*

Median QMG score at admission was 18 points (range: 12–25), median increase was 12 points (range: 7–24). Patients received either plasma exchange (seven patients) or intravenous immunoglobulins (IVIG; five patients). Two patients received IVIG after plasma exchange due to insufficient clinical improvement.

**Fig. 1:**
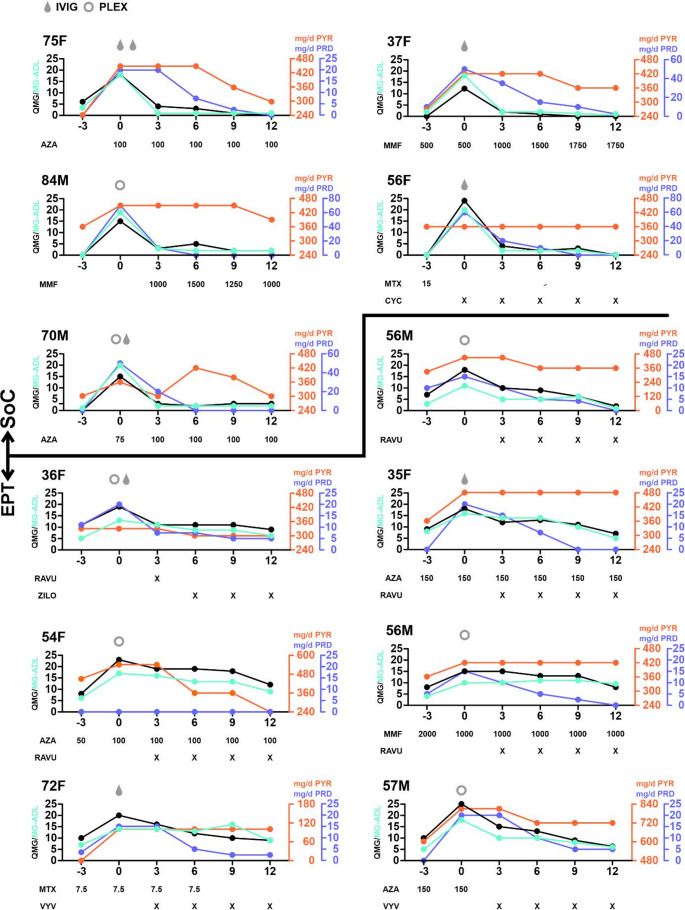
Disease course of following myasthenic exacerbation (ME) Time is shown in months; “0” indicates onset of ME. Age and gender of the patient are indicated in the upper left corner of the respective graph. The first five patients received standard-of-care treatment whereas the other seven patients underwent endplate protecting treatment. Abbreviations: SoC: standard-of-care; EPT: endplate protecting treatment; IVIG: intravenous immunoglobulins; PLEX: plasma exchange; QMG: quantitative myasthenia gravis score; MG-ADL: myasthenia gravis activities of daily living questionnaire; AZA: azathioprine, MMF: mycophenolate; MTX: methotrexate; CYC: cyclophosphamide; PYR: pyridostigmin; PRD: prednisolone; RAVU: ravulizumab; ZILO: zilocuplan; VYV: efgartigimod alfa

Following discharge, two recovery patterns were observed. Five patients recovered baseline QMG scores, achieved PASS at month three, and thus remained on SoC (Fig. 1; upper part).

Seven patients had persistent, severe symptoms and required EPT (C5I: five; FcRnI: two). Notably, compared to the SOC group, these patients showed higher body weights (75 kg (range 57–170) vs. 65 kg (range 58–82)) and higher steroid exposure at baseline (57% vs. 20%). None of the patients developed another ME during follow-up.

These patients are shown in Figure 1 (lower part).

Among EPT patients, we observed improvement of both MG-ADL and QMG scores at month 12. However, recovery was substantially prolonged. Only one patient deemed his symptoms acceptable at month six EPT (median QMG improvement from induction: 3 points (range: 0–7)). However, further improvement was seen at month nine when 4/7 patients reported PASS (median QMG improvement from induction: 8 points (range: 2–9)). Generally, MG-ADL scores closely resembled QMG scores in both SOC and EPT patients.

## Discussion

We here present data of a highly selected cohort of seropositive MG patients and their disease courses over 12 months following ME. While sequelae of ME resolved within three months in some patients, more than half suffer ongoing disability within a year. This aligns with prior analyses of ME patients which was here expanded to explicitly assess EPT patients as well [2]. Notably, disability persists for months despite rescue therapy and subsequent and rapid escalation to EPT.

This delayed response contradicts results from randomized trials only at a first glance. Randomized trials have uniformly shown rapid treatment responses following induction of both FcRnI and C5I and trials yielded significant benefits despite short durations ranging down to 12 weeks in one trial [4–7]. However, patients with recent ME (such as ours) were excluded from these studies and thus, nothing is known on whether EPT results in accelerated recovery.

A larger real-world study confirmed the rapid onset of EPT and showed significant clinical improvements in month six but nothing was known on the proportion of patients with recent ME [8]. Of note, another real-world study showed only modest rapid effects of both C5I and FcRnI in the real-world setting [9]. The proportion of patients with recent ME was neither stated here.

However, as demonstrated in this study, some patients in the real-world setting take more time to achieve a clinical response. This highlights the need for a sufficiently long course of treatment in order to truly assess the full effectiveness of the medication also of C5 inhibitors and FcRn modulators especially in patients, who are not only refractory to standard of care therapy, but who also experienced ME. All our patients required rescue therapy for ME and this is suggestive of increased endplate-degradation at baseline. Thus, their recovery might depend on endplate recovery as well.

Our study has some limitations including its observational character, varying doses of pyridostigmine or corticosteroids, and unknown confounders potentially driving treatment decisions and responses. Moreover, factors possibly influencing the outcome after ME like older age as well as longer disease duration of SOC patients, potentially explaining a less active disease. As already mentioned above, the median body weight of EPT patients was higher as well. Patients in the EPT group were more often treated with corticosteroids and one patient suffered from a thymoma before TE as possible correlate for higher disease activity.

Nonetheless, we included a highly selected cohort of patients having undergone early thymectomy following diagnosis. The average peak QMG score in our group was 18, not much different from patients in clinical trials. Furthermore, we provide detailed outcome measures, as our patients are prospectively evaluated in our specialized MG centre.

Overall, our study underlines that recovery from ME as surrogate for uncontrolled MG can be prolonged, despite rescue therapy and administration of new treatments.

This should prompt physicians not to judge the treatment response of EPT too early in the real-world setting despite the repeatedly shown rapid onset of an effect within clinical trials.

## Data Availability

Anonymized patient data will be shared upon reasonable request from qualified investigators.
